# Parasitological detection of *Theileria* parasites in livestock in various regions of the Northern Emirates of the United Arab Emirates

**DOI:** 10.14202/vetworld.2023.1071-1074

**Published:** 2023-05-17

**Authors:** Sameera M. A. Ismaeil, Rajeesh K. T. Parambil, Manal S. Kerab, Ali M. K. ElBakri, Ideisan I. Abu-Abdoun

**Affiliations:** 1 Ministry of Climate Change and Environment, Veterinary Laboratory Section, PO Box 926, Sharjah, United Arab Emirates; 2 Department of Medical Laboratory Sciences, College of Health Sciences, PO Box 27272, University of Sharjah, Sharjah, United Arab Emirates; 3 Department of Chemistry, College of Science, PO Box 27272, University of Sharjah, Sharjah, United Arab Emirates

**Keywords:** microscopic examination, *Theileria*, UAE

## Abstract

**Background and Aim:**

Theileriosis, caused by tick-borne hemoprotozoans of the genus *Theileria*, severely impacts the economics of the livestock industry in most tropical and subtropical countries. The aim of the present study was to detect *Theileria* spp. in domesticated animals (camels, cows, sheep, and goats) using direct microscopy and to determine the infection rate in geographically distinct regions in the northern emirates of the UAE.

**Materials and Methods:**

Blood samples (n = 536) were collected from clinically symptomatic and asymptomatic domesticated animals and subjected to Giemsa staining and examined microscopically for the identification of *Theileria*.

**Results:**

Smears showed an overall rate of positivity for *Theileria* spp. in 325/536 (60.6%) animals. Different infection rates were recorded across the various animal groups in the different study areas (Middle region 215/386 [55.7%], East region 100/139 [71.9%]). Of the 11 goat samples collected from the North region, 10 (90%) were positive. Infection rates per animal group based on microscopy were as follows: camels, 3/35 (8.5%); cows, 19/36 (52.7%); goats, 200/303 (66%); and sheep, 103/162 (63.5%). Real-time polymerase chain reaction confirmation of all microscopy-positive samples identified 23/325 (7.1%) results as false-positive.

**Conclusion:**

This study clarified that *Theileria* spp. is present in the Middle (Sharjah, Umm Al Quwain, and Ajman), East, and North regions. This report also confirmed the use of direct microscopy with Giemsa-stained blood films as the method of choice for diagnosing acute infections. Further work is needed to molecularly determine the prevalence and species of *Theileria* spp. circulating in the different parts of the UAE.

## Introduction

*Theileria* species are blood protozoa transmitted by Ixodidae ticks. Pathogenic species cause theileriosis in camels, cattle, and small domestic ruminants, frequently causing death and illness, thus adversely impacting the health of the livestock and the economics of the livestock industry [1]. Reported clinical signs include jaundice, tachycardia, weakness, fever, reduced milk production, and abortion/stillbirth as valid clinical signs for the clinical diagnosis of theileriosis [1].

There are various techniques available for detecting and identifying *Theileria* spp. in clinical samples. Microscopy has always been the main tool for diagnosing *Theileria*, especially in animals demonstrating clinical symptoms [2]. The emergence of molecular techniques such as conventional PCR and real-time PCR has led to the accurate detection and identification at the species level of *Theileria* spp., providing precise diagnosis and meticulous prevalence data in many countries [3].

This study aimed to detect *Theileria* spp. in clinically symptomatic farm animals (camels, cows, sheep, and goats) using Giemsa-stained blood films and to determine the infection rate in geographically distinct regions in the northern emirates of the UAE.

## Materials and Methods

### Ethical approval

No ethical approval was required for this study as all blood samples were routinely collected for official diagnostic and monitoring purposes and subsequently made available for this study. The study was conducted in accordance with the current UAE and European guidelines on animal protection.

### Study period and location

**Figure-1:**
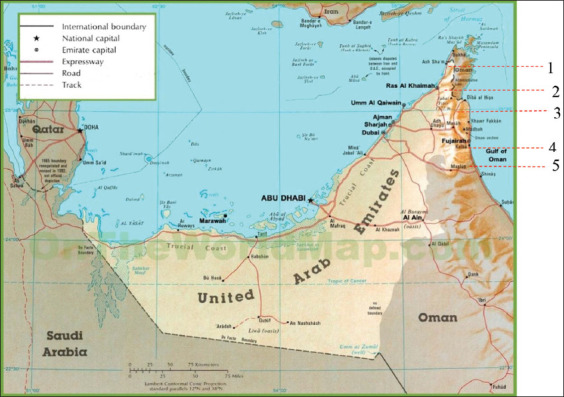
Map of United Arab Emirates showing the areas where samples were collected: 1. Shammal (Northern region), 2. Digdaga (Northern region), 3. Dibba (Eastern region), 4. Kalba (Eastern region), 5. Masafi (Eastern region) [Source: https://ontheworldmap.com/uae/uae-road-map.html].

The study was conducted from January to April 2020. Samples were collected from three different regions of the northern emirates of the UAE: Middle region (Sharjah, Umm Al Quwain, and Ajman); East region (Kalba, Dibba, Masafi, and Fujairah); and North region (Digdaga and Shamal) (Figure-1). A total of 536 blood samples were collected from clinically symptomatic (526 samples) and asymptomatic (ten samples) farm animals between January and April 2020. The symptomatic animals showed signs typical of theileriosis. Reported clinical signs included fever, anemia, emaciation, and weight loss. Blood smears were prepared and examined to confirm the clinical diagnosis of both diseased and asymptomatic animals. Table-1 shows the animals involved in the study.

**Table-1:** Study samples and regions.

Host	Middle Region	East Region	North Region	No. of animal species
		
	No. of samples	No. of samples	No. of samples	
Camel	30	5	0	35
Cow	36	0	0	36
Goat	170	122	11	303
Sheep	150	12	0	162
Total per region	386	139	11	
Total	536			

### Collection of samples and microscopic examination

Under the supervision of a veterinarian, 3 mL of blood samples were collected in ethylenediaminetetraacetic acid (EDTA) tubes from the suspected animals. Thin smears were prepared from EDTA whole blood on clean and dry slides, stained using KIT RAL 555 as per the manufacturer’s instructions, and microscopically examined for the detection of *Theileria* spp. at 100× objective magnification. An average of 12 microscopic fields was examined before a sample was considered negative. A sample was reported to be microscopically positive on confirmation by two technicians. Real-time PCR was performed on microscopically positive samples only.

## Results and Discussion

The present study showed an overall rate of positivity for *Theileria* spp. in 325/536 (60.6%) animals by direct microscopy using Giemsa-stained thin blood films (Table-2). The following positivity rates were recorded across the various animal groups according to the study regions. The Middle region revealed a positivity rate of 215/386 (55.7%), with each animal group showing the following positive rates: camels, 2/30 (6.6%); cows, 19/36 (52.77%); goats, 100/170 (58.82%); and sheep, 94/150 (62.66%). Samples from the East region revealed an overall positivity rate of 100/139 (71.9%), including 1/5 (20%) camels, 90/122 (73.3%) goats, and 9/12 (75%) sheep. Of the 11 goat samples collected from the North region, 10/11 (90%) were positive.

**Table-2:** Total infection rates of *Theileria* spp. in the study animals.

Host	Number of Animals in Middle Region	Number of Animals in East Region	Number of Animals in North Region	Number of positive samples (infection rates) (%)
Camel	30	5	0	3/35 (8.5)
Cow	36	0	0	19/36 (52.7)
Goat	170	122	11	200/303 (66)
Sheep	150	12	0	103/162 (63.5)
Total	386	139	11	325/536 (60.6)

Infection rates per animal group based on microscopy in the present study were as follows: camels, 3/35 (8.5%); cows, 19/36 (52.7%); goats, 200/303 (66%); and sheep, 103/162 (63.5%). Real-time polymerase chain reaction (Real-time-PCR) confirmation of all microscopy-positive samples identified 23/325 (7.1%) as false-positive (data not shown).

Theileriosis transmitted by various tick vectors of the family *Ixodidae*, infects a wide range of animals, both wild and domesticated [4]. This study demonstrated that *Theileria* spp. is present in the examined animals. The diagnosis of *Theileria* spp. is normally based on clinical signs and the demonstration of protozoa in stained blood smears [5]. Nevertheless, this method is unreliable in the case of chronic infections. The present report revealed an overall positivity rate of 60.6% in animals from all of the study regions (Table-2). Similar results were described from a study in Egypt using Giemsa-stained blood smears, where a prevalence rate of 65.4% was reported [6]. However, our results contrast with those reported by several groups who described various prevalence rates in cattle using blood smears [1, 7–9]. In a study comparing the microscopic and molecular detection of *Theileria annulata* infection in cattle in Egypt, the prevalence of bovine theileriosis was 9.31%, as determined by microscopic examination of Giemsa-stained thin blood smears [1]. The same study also revealed a prevalence rate of 11.4% using PCR. Interestingly, most of the clinically positive animals and all of those positive as determined by microscopic examination and PCR were found to have ticks similar to those found in the El-Wadi El-Gadid province of Egypt [1].

It is worth mentioning that, in our veterinary laboratory, all microscopically positive samples need to undergo confirmatory analysis by real-time PCR; the real-time PCR results indicated that 302 samples were true-positive in the current study, while 23 were false-positive. Although a negative microscopy result does not definitively rule out infection, a limitation of our study was that there was no confirmation by real-time PCR. Therefore, conducting a comparative study of the two techniques was impossible.

Limited studies have investigated rates of infection with *Theileria* spp. in the UAE. A survey by Jaffer *et al*. [3] indicated that horses were infected with *Theileria equi* at a prevalence rate of 32.4%. In addition, Al-Deeb *et al*. [10] reported for the first time the presence of *Theileria annulata* in *Hyalomma dromedarii* ticks in the United Arab Emirates. More recently, a cross-sectional DNA-based study assessed the presence and prevalence of *Theileria* among other microbes in ticks infesting livestock (camels, cows, sheep, and goats) in Abu Dhabi, Dubai, and Sharjah using PCR. *Theileria* spp. was found at a low rate only in *Hyalomma anatolicum* ticks collected from cows and goats in Sharjah [11].

The current report revealed an overall rate of positivity for *Theileria* spp. of 55.7% in animals from the Middle region (Sharjah, Um Al Quwain, and Ajman emirates). To the best of our knowledge, this is the first study describing this finding in these three emirates. Unlike in the study conducted by Perveen *et al*. [11], *Theileria* spp. was detected in Sharjah, Umm Al Quwain, and Ajman, with the different animal groups showing the following positivity rates: camels (6.6%), cows (52.77%), goats, (58.82%), and sheep (62.66%). The detection of *Theileria* spp. in cows and goats from Sharjah is interesting. It coincides with the presence of *T. annulata* and *Theileria ovis* in ticks collected from cows (4.5%) and goats (10%), respectively, in Sharjah study by Perveen *et al*. [11]. Moreover, a very high infection rate was noted in sheep in Sharjah (62.66%), unlike in Abu Dhabi and Dubai, where none of the ticks examined for *Theileria* was positive. Samples from the East region (Kalba, Dibba, Masafi, and Fujairah) included smaller numbers of camels (five samples) and sheep (12 samples) to warrant a clear picture of the infection rates in these two animal groups. These low rates contrast with that for goats (122 samples), in which 73.3% were positive. In this context, it is currently not possible to determine the epidemiology of *Theileria* among other animals from the East region.

Overall, infection rates per animal group based on microscopy were as follows: camels (8.5%), cows (52.7%), goats (66%), and sheep (63.5%). A study in six regions of Saudi Arabia reported much lower rates of infection with *Theileria* spp. in the same animal hosts as those in the current study [12]. It is worth stating that the Saudi study investigated the presence of these parasites from randomly selected animals, while our samples were mostly from animals with suspicious clinical signs. Further work is needed to determine molecularly the prevalence of such infection and identify the species circulating in the different regions of the UAE.

## Conclusion

The present study clarified that *Theileria* spp. is present in the Middle (Sharjah, Umm Al Quwain, and Ajman), East, and North regions of the United Arab Emirates in the studied animal hosts. We have shown that only 23 samples (7.1%) were false-positive, as confirmed by real-time PCR; nonetheless, the present study also confirmed the value of direct microscopy on Giemsa-stained blood films as the method of choice in acute infections. On the other hand, it is imperative that all samples negative by microscopy also undergo confirmatory analysis by PCR to detect false-negatives in cases in which livestock are asymptomatic or human error which has occurred due to a lack of experience.

## Authors’ Contributions

SMAI: Designed the study, experimental work, data analysis, investigation, and drafted and revised the manuscript. MSK: Literature search and drafted the manuscript. RKTP: Performed the study, data analysis, and drafted and revised the manuscript. AMKE: Conceptualization, managed the discussion, and drafted and revised the manuscript. IIA: Investigation, coordination, and review and editing. All authors have read, reviewed, and approved the final manuscript
